# The Application of the Nanofiltration Membrane NF270 for Separation of Fermentation Broths

**DOI:** 10.3390/membranes12121263

**Published:** 2022-12-14

**Authors:** Wirginia Tomczak

**Affiliations:** Faculty of Chemical Technology and Engineering, Bydgoszcz University of Science and Technology, 3 Seminaryjna Street, 85-326 Bydgoszcz, Poland; tomczak.wirginia@gmail.com

**Keywords:** dielectric exclusion, Donnan exclusion, fermentation broth, nanofiltration, organic acid, polyamide membrane, pumping effect, screening effect, steric exclusion

## Abstract

The potential for nanofiltration (NF) in removing both relatively low molecular weight (MW) organic species and charged solutes from complex media is noteworthy. The main aim of the current work was to improve understanding of the separation mechanisms of fermentation broths components in the NF process. For this purpose, the experimental investigations were performed using the commercial polyamide NF270 membrane. The feed solution was ultrafiltered 1,3-propanediol (1,3-PD) broths. The separation results were analyzed and discussed in light of the detailed characteristics of both the membrane and the broth components. It has been noted that the membrane ensured the complete 1,3-PD permeability and significant rejection of some feed components. A thorough analysis showed that the retention of carboxylic acids was based on both the Donnan effect and sieve mechanism, according to the following order: succinic acid > lactic acid > acetic acid > formic acid. Indeed, acids retention increased with increasing charged acids ions valency, Stokes radius (r_S_) as well as MW, and decreasing diffusion coefficient (D). In turn, for ions, the following orders retention was determined: SO_4_^2−^ = PO_4_^3−^ > Cl^−^ and Ca^2+^ > Na^+^ > NH_4_^+^ ~ K^+^. It indicated that the ions retention increased with increasing ions charge density, hydrated radius (r_H_), and hydration energy (E_h_). It showed that the separation of the ions was based on the Donnan exclusion, sieving effect, and dielectric exclusion.

## 1. Introduction

Nowadays, 1,3-propanediol (1,3-PD) is considered one of the most important value-added chemicals which has been widely used in the polymer, pharmaceutical, and cosmetics industries. On an industrial scale, 1,3-PD is manufactured mainly from fossil-derived chemicals via Shell or Degussa processes [1,2,3]. However, the synthesis of 1,3-PD by bioconversion would be a step toward achieving sustainable development goals [4]. Indeed, the production of 1,3-PD by fermentation is an environmentally friendly and energy-saving alternative to traditional chemical synthesis [5].

It is apparent that post-fermentation solutions are characterized by very complex compositions. Generally, they consist of biomass residues, inorganic salts, and organic compounds, such as organic acids. Consequently, obtaining a high-purity desired product from such complex media requires integrated and multi-stage purification processes [6,7]. For this purpose, various membrane techniques can be used. It has been widely documented that the complete retention of microbial cells, proteins, and other molecules can be achieved by microfiltration (MF) [8,9,10] and ultrafiltration (UF) [11,12,13] processes. However, most organic compounds and salts present in fermentation broths have a low molecular weight (MW), hence, they may not be rejected effectively and sufficiently by the above-mentioned techniques.

Nanofiltration (NF) is a pressure-driven membrane process that has the unique capability to remove both relatively low MW organic species and charged solutes. NF is a very effective, environmentally friendly, energy-efficient, and simple technology [14,15,16,17]. NF membranes have a microporous structure with a pore diameter of less than 2 nm and a molecular weight cut-off in the range from 200 to 1000 Da [15,18,19]. Commercial NF membranes are thin-film composites made mainly of polyamide (PA), polypiperazineamide, cellulose acetate (CA), and polyethersulfone (PES) [20,21,22]. Generally, NF membranes are neutral or negatively charged materials [23,24]. Notwithstanding, the feed pH may affect the nature of the membrane surface charge due to the disassociation of functional groups. For instance, an active layer made of PA contains both amine (R-NH_2_) and carboxylic (R-COOH) functional groups which may ionize in an aqueous solution [25]. Indeed, at low pH, the group R-NH_2_ protonates and changes the charge to a positive one R-NH_3_^+^, while at high pH, the group R-COOH deprotonates and acquires a negative charge R-COO^−^ [26].

**Figure 1:**
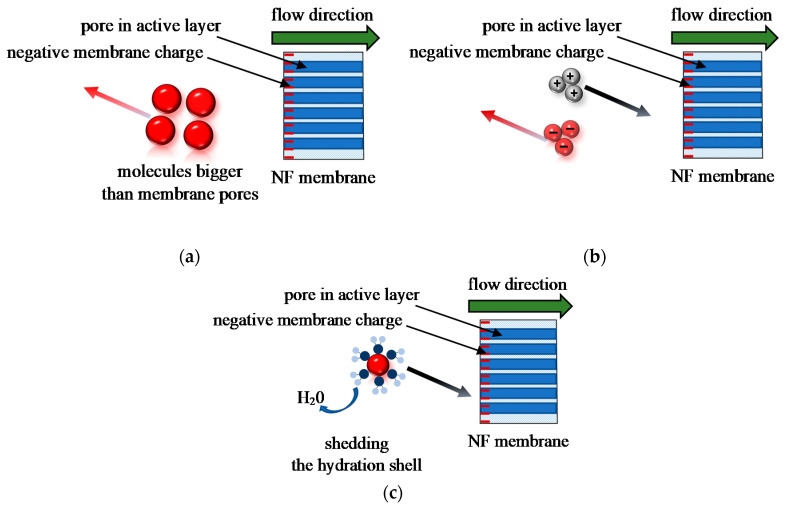
Mechanisms of solute separation in NF: (**a**) Steric exclusion; (**b**) Donnan exclusion; (**c**) Dielectric exclusion.

The separation selectivity of NF membranes is mainly attributed to three following mechanisms: size exclusion (size screening), Donnan exclusion (electrostatic repulsion), and dielectric exclusion. For the nanofiltration of a neutral solute, the retention is fixed according to the size exclusion (Figure 1a), while electrostatic interactions are negligible. Evidently, the molecules with sizes larger than that of the membrane pores are rejected. In turn, the transport of solutes with a size close to or smaller than the size of pores involves first partitioning of the solutes into the membrane and then, diffusion through the pores [27]. Hence, the membrane pores size and molecular dimensions of solutes are key factors affecting the rejection during the NF process run. In turn, the retention is independent of solution concentration and pH [28]. Importantly, membrane pore radii are constant unless the membrane is subjected to significant changes in the process conditions that may lead to pore swelling [29,30]. For instance, according to Dang et al. [31], the feed temperature affects the pores radii of the NF270 membrane. Indeed, the above-mentioned authors have demonstrated that increasing the temperature from 20 to 40 °C leads to an increase in the average pore radius from 0.39 to 0.44 nm.

In turn, the separation of charged molecules is typically based on a combination of size exclusion and electrical effects. In the aqueous solutions, ions exist as hydrated ions with solvation shells, hence, the size-selectivity of the membrane is achieved by the difference in the size of the membrane pores and hydrated ions. The repulsion of the ions from the membrane surface is described by the Donnan exclusion (Figure 1b). If a charged membrane is put in contact with an ionic solution, ions with the same charge sign as that of the membrane are excluded, while the ions with the opposite charge sign pass the membrane. Consequently, negatively charged NF membranes provide high rejection of solutes with high anionic charge density. It has been documented that the rejection of charged solutes by NF membranes depends on the feed concentration and the charge of the membrane [29], while is not affected by the changes in the membranes pore size [31].

Another important mechanism that should be considered is dielectric exclusion (Figure 1c). Although dielectric exclusion has been widely discussed over the years [32], in the recently published review papers [16,20,33] it has been pointed out that it still is not a well-understood phenomenon. In short, the mechanism of dielectric exclusion is associated with the solute resistance to penetrate the membrane caused by the energy barrier due to the shedding of the hydration shell [32]. Indeed, when ions pass the membrane through the pores having a size equal to or smaller than ion hydrated size, ions dehydration is required [34,35]. Consequently, according to the ion dehydration theory, lower ion hydration energy (E_h_), defined as the energy released during the ion hydration process [36,37], can enhance steric exclusion. It is due to the fact that in this case, the water shells surrounding the ion are easily removed during the flow through the membrane [27,38,39]. It has been claimed that, contrary to the Donnan effect, the mechanism of dielectric exclusion is independent of the ion sign [34].

**Figure 2:**
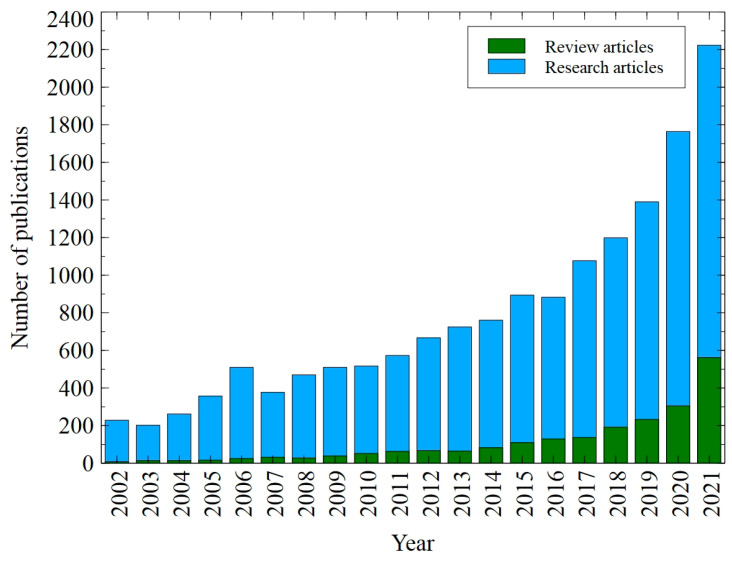
The number of papers focused on nanofiltration according to Science Direct. Keywords: nanofiltration + fermentation broth, data retrieved: 11 October 2022.

After all, it should be noticed that although NF is a relatively new process among pressure-driven membrane techniques [40], it has received increasing attention from researchers. Indeed, a thoroughly performed literature review allowed us to demonstrate the significant increase in the number of both review and research articles on the NF process published in the last five years (Figure 2). Moreover, NF has increasingly attracted a wide range of applications. Briefly, the literature findings reveal that NF has been successfully used for the treatment of drinking water [41,42,43], wastewater reuse [44,45,46], and food processing [47,48,49]. In addition, recent years have witnessed a growing interest in applying the NF to separate various components from actual and synthetic fermentation broths [6,7,24,29,50,51,52,53,54,55,56,57,58,59,60,61,62,63,64,65,66]. For instance, Antczak et al. [7] investigated the separation of succinic acid from fermentation broth with the use of a flat sheet NF membrane made of polyamide (active layer) and polysulfone. The above-mentioned authors have demonstrated that the used membrane ensured 92% of succinates rejection and partial removal of other compounds, such as glycerol, lactose, and monocarboxylic acids. A study conducted by Li et al. [58] analyzed the separation of L-glutamine from fermentation broth. For this purpose, a commercial polymeric NF membrane composed of a sulfonated polyethersulfone layer was used. It has been found that the NF allowed to effectively separate l-glutamine and l-glutamate from the feed. Furthermore, it has been noted that the separation performance was strongly affected by the pH, transmembrane pressure (TMP), and broth concentration. In turn, in a recently published research paper [62] it has been clearly demonstrated that NF is an effective strategy to purify xylitol obtained by fermentation of sugarcane bagasse hemicellulose hydrolysate.

Zaman et al. [56] have indicated that more investigations are needed to validate the performance of commercial NF membranes in particular applications. Accordingly, to the best of the author’s knowledge, only a few studies have been carried out on the use of NF membranes for the separation of 1,3-PD fermentation broths. In addition, summarizing the literature data, it can be concluded that the NF process has not been yet fully evaluated. Indeed, despite a large volume of research, it still has to grow in terms of understanding the separation mechanism of complex solutions.

Consequently, the main aim of the current work was to improve understanding of the separation mechanisms of 1,3-PD fermentation broths components in the NF process. For this purpose, an experimental investigation was carried out with actual broths obtained via bioconversion of glycerol. The studies were performed with the use of commercial flat-sheet thin-film polyamide NF270 nanofiltration membrane. The separation results were analyzed and discussed in light of the detailed characteristics of both the membrane and the broth components.

## 2. Materials and Methods

### 2.1. Feed Solution

In the present study, the fermentation broths obtained via bioconversion of glycerol to 1,3-propanediol were used as a feed. Prior to NF, in order to remove bacteria and high molar mass compounds from the solutions, the UF process of broths was performed. As a result, the solutions were sterile and their turbidity was equal to about 1 NTU. The composition of the fermentation broths and the characteristics of each compound are given in Table 1. 1,3-PD was the main product, while mono-carboxylic acids (lactic, acetic, and formic acid) and dicarboxylic acid (succinic acid), as well as ethanol, were the main by-products. The feed pH was 7. In order to ensure the simplicity of the process and to reduce the overall costs, the NF was conducted without the use of a pH buffer. The NF experiments were carried out three times. For each process run, a new portion of the feed solution was used.

### 2.2. NF Set-Up

**Figure 3:**
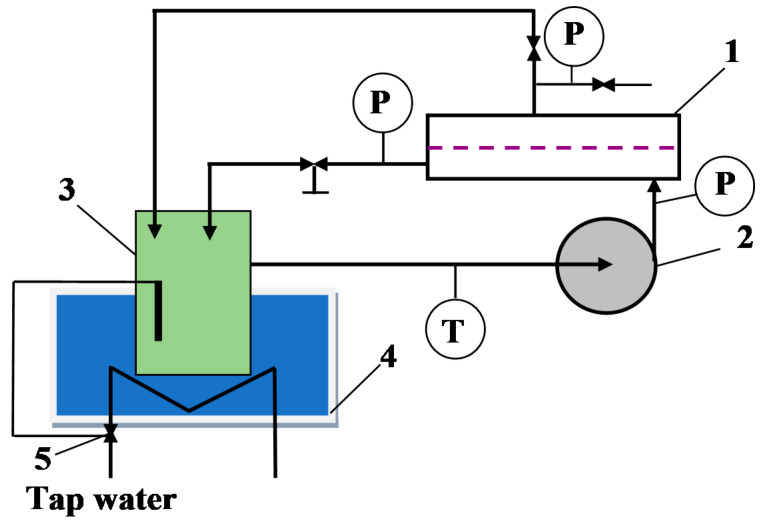
Experimental NF set-up. 1—SEPA-CFII module, 2—pump, 3—feed tank, 4—cooling bath, 5—thermostatic Grundfos valve, P—manometer, T—thermometer.

Nanofiltration experiments were conducted using a laboratory-scale filtration system (Figure 3). It was equipped with the following main parts:

(i) Sepa-CFII plate membrane module manufactured by GE Osmonics (Minnetonka, MN, USA) with the membrane active area equal to 0.015 m^2^; the channel in the module was filled with polypropylene net-spacer (50 mesh),
(ii) 3CP Stainless Steel Plunger Pump model 3CP1221 (CAT PUMPS, Hampshire, England),
(iii) feed tank (volume 5 L),
(iv) cooling bath (thermostatic Grundfos valve).

The process was carried out under controlled operational parameters. The feed temperature and TMP were equal to 298 K and 1.4 MPa, respectively. The feed flow rate of 10 L/min was applied. The permeate and retentate samples were collected at 60 min intervals.

**Figure 4:**
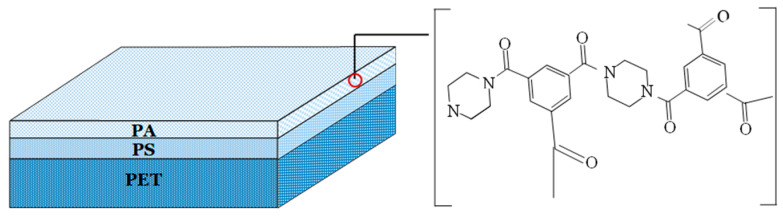
Structure of NF270 membrane. PA-polyamide, PS-polysulfone, PET-polyester.

In the present study, the commercial flat-sheet thin-film polyamide NF270 nanofiltration membrane from DOW-Filmtec (Minneapolis, USA) was used. It is composed of the active layer made from semiaromatic piperazine-based PA and the support layers from polysulfone (PS) and nonwoven polyester (PET) (Figure 4). Table 2 summarizes the physicochemical characteristics of the membrane, as given by the manufacturer, and several studies reported in the literature [31,33,78,79,80,81,82,83,84,85,86]. During the investigation performed in the present study, the membrane surface was negatively charged since the pH of the fermentation broths (equal to 7) was higher than the isoelectric point of the membrane (equal to 4).

### 2.3. Experimental Protocol

Membrane performance was characterized by the following parameters: permeate flux, normalized permeate flux, permeability, hydraulic resistance, and retention. The permeate flux (J) was defined, as follows:

(1) $$J=\frac{V_{P}}{S\cdot t}$$

where V_P_ [L] is the volume of permeate, S [m^2^] is the active surface of the membrane and t [h] is the unit of time.

The normalized permeate flux (J_r_) was defined as a ratio of the actual permeate flux (J_i_) and initial distilled water flux (J_0_):

(2) $$J_{r}=\frac{J_{i}}{J_{0}}$$

The membrane permeability (L_p_) was calculated from the linear regression of the distilled water flux vs. TMP:

(3) $$L_{p}=\frac{J_{0}}{\mathrm{TMP}}$$

The hydraulic resistance of the clean membrane (R_m_) was determined according to Darcy’s law:

(4) $$R_{m}=\frac{\mathrm{TMP}}{{µ}_{0}J_{0}}$$

where µ_0_ [Pa·s] is the viscosity of water at a temperature equal to 298 K.

The total membrane resistance (R_T_) was calculated, as follows:

(5) $$R_{T}=\frac{\mathrm{TMP}}{{µ}_{p}J}$$

where µ_p_ [Pa·s] is the viscosity of permeate solution at a temperature equal to 298 K.

The membrane efficiency was characterized in terms of separation efficiency expressed by the rejection (R_i_) of a given compound given by the following equation:

(6) $$R_{i}=\left(1-\frac{C_{i,\;P}}{C_{i,R}}\right)\cdot 100\%$$

where C_i,P_ [g/dm^3^] and C_i,R_ [g/dm^3^] are the concentrations of each species in the permeate and in the concentrate, respectively.

Once the NF experiment run was completed, a membrane cleaning procedure was applied. For this purpose, firstly, the membrane was rinsed with distilled water under a TMP of 0.05 MPa and a temperature equal to 298 K for 2 h. Afterward, the membrane was soaked in distilled water for 12 h (osmotic rinsing).

### 2.4. Analytical Methods

The fermentation broths and the permeate obtained by the NF process were characterized in terms of composition, turbidity, and viscosity. The concentrations of glycerol, 1,3-PD, and by-products were determined by high-performance liquid chromatography HPLC using a UlitiMate 3000 (Thermo Fisher Scientific, Germering, Germany). Determination of anions and cations in the solutions was performed using an 850 Professional IC ion chromatograph (Herisau Metrohm AG, Herisau, Switzerland). The turbidity of the feed and permeate was analyzed using a HACH (Hach Company, Loveland, CO, USA) turbidimeter (2100ANIS). Viscosity was measured with the use of a viscometer (BROOKFIELD DV-II + Pro) with a UL Adapter (BROOKFIELD ENGINEERING LABORATORIES, Middleboro, MA, USA). Details of the above-mentioned analytical methods were presented in previous studies [13,87].

## 3. Results and Discussion

### 3.1. Membrane Performance

**Figure 5:**
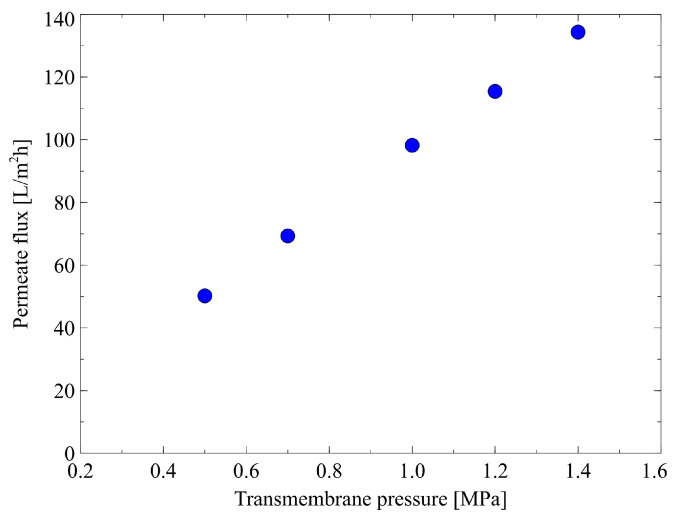
Water flux as a function of transmembrane pressure. T = 298 K.

The hydraulic properties of the NF270 membrane were determined by performing the filtration tests with the use of distilled water at a temperature of 298 K and TMP in the range from 0.5 to 1.4 MPa (Figure 5). It has been determined that at a TMP of 0.5 MPa, the permeate flux was equal to 50.22 L/m^2^h. As expected, the flux linearly increased with the applied TMP. Consequently, at TMP of 0.7, 1.0, 1.2, and 1.4 MPa, the flux was equal to 69.33, 98.22, 115.42, and 134.31 L/m^2^h, respectively. Obviously, this observation is related to the fact that the pressure is the driving force for permeate flux in the NF process, according to the following equation:

(7) $$J=L_{p}\left(\mathrm{TMP}-\Delta \pi \right)$$

where Δπ [Pa] is the osmotic pressure difference across the membrane.

It has been noted that the membrane permeability L_p_ was equal to 9.8 L/m^2^h bar. It is important to underline that this finding is similar to the results reported in other studies [44,86,88]. Therefore, it can be indicated that the membrane used in the present study is a type of “loose” NF membrane.

Using Equation (4), the membrane hydraulic resistance was found to be 5.49 × 10^13^ m^−1^. Worthy of note, the same order of R_m_ (7.4 × 10^13^ m^−1^) was noted for a nanofiltration DK membrane made of polyamide (active layer) and polysulfone supplied by GE Osmonics [71].

**Figure 6:**
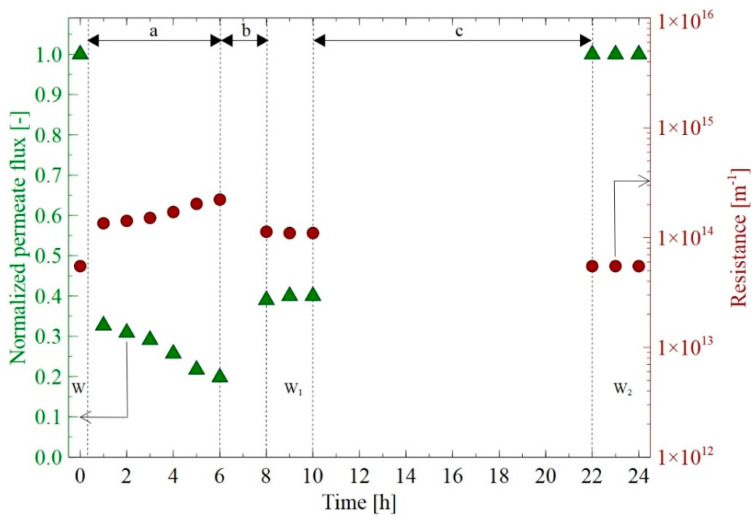
Changes in normalized permeate flux during the NF membrane run. a—NF of the fermentation broth, b—membrane rinsing, c—membrane soaking, W_1_, W_2_—measurements for distilled water.

The normalized permeate flux J_r_ for all stages in the 24-h run of the NF membrane is presented in Figure 6. Stage “a” (6 h) presents the flux noted during the separation of 1,3-PD fermentation broth. As expected, the permeate flux decreased sharply at the beginning of the process run. Indeed, during the first hour, the normalized flux of 0.32 was noted, corresponding to the total membrane resistance R_T_ of 1.35∙10^14^ m^−1^. In the following hours, a systematic reduction in the membrane performance and an increase in the total resistance were noted. Finally, after 6 h, the permeate flux of 26.66 L/m^2^h was obtained which contributed 20% of the initial flux. At the end of the process, the total resistance was equal to 2.22∙10^14^ m^−1^, indicating the ratio R_T_/R_m_ equal to 3. This finding points out that during the separation of fermentation broth, the total resistance was three times higher than that determined for the clean membrane R_m_.

In the literature, there is general agreement that the observed phenomenon is typical for membrane processes. Furthermore, flux decline during the separation of fermentation broths with the use of various NF membranes has been widely reported in previous studies [7,50,57,62,63,65,66]. For instance, Thuy et al. [65] noted that in the first few minutes of separation of clarified succinic fermentation broth with the use of a spiral-wound NF membrane, the permeate flux decreased from 1.22 L/m^2^h to 0.2 L/m^2^h. The above-mentioned authors have shown that after 21 h of the NF run, the flux was equal to 0.09 L/m^2^h which corresponded to a 92.6% reduction in comparison to the initial flux. In turn, Alves et al. [62] during the separation of xylitol from lignocellulosic biomass, noted the normalized flux equal to 73.7, 48.7, and 53.9% for the NF (polypiperazine amide), NP010, and NP030 (polyether-sulfones) membranes, respectively.

From a theoretical point of view, it is recognized that the noted decline in the membrane performance could be attributed to several phenomena, including concentration polarisation, species adsorption on the membrane surface, and formation of cake and gel formation. According to Antczak et al. [7], flux decline during the NF of fermentation broths can be related to the fouling caused by the presence in the broth of charged cations, which are more readily adsorbed on the membrane surface than anions.

After the NF process ran, the membrane cleaning was performed. For this purpose, the membrane was rinsed with distilled water (Figure 6, stage “b”) for 2 h, and subsequently, the membrane was soaked for 12 h (Figure 6, stage “c”). It has been demonstrated that the proposed method of membrane cleaning has been effective. Indeed, the membrane rinsing allowed us to obtain the normalized permeate flux of 0.4 that corresponded to the transfer resistance equal to 1.1·10^14^ m^−1^ (Figure 6, “W_1_”). In turn, after the membrane soaking in distilled water, the complete membrane performance has been recovered (Figure 6, “W_2_”). Based on the findings presented above, it can be indicated the irreversible fouling was minimal under the process parameters applied in the present study.

### 3.2. Separation of Organic Compounds

#### 3.2.1. 1,3-Propanediol and Glycerol

**Figure 7:**
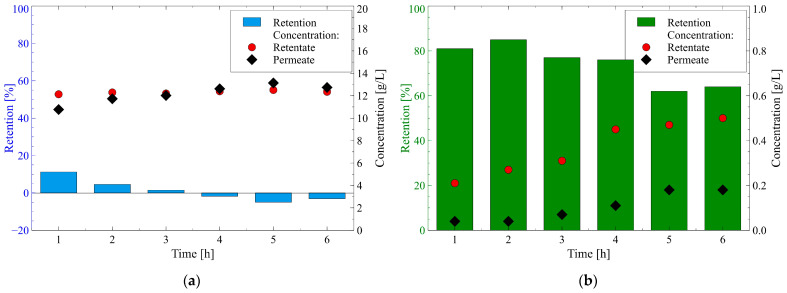
Changes in retention and concentration of the target product and substrate in the retentate and permeate during the NF process of fermentation broth: (**a**) 1,3-propanediol; (**b**) Glycerol. TMP = 1.4 MPa, T = 298 K.

With regard to the separation of organic electrolytes by the NF process, the dissociation equilibrium is of great importance [24]. Since the pKa of 1,3-PD and glycerol (14.46 and 14.40, respectively, Table 1) is much higher than the pH of the fermentation broth (equal to 7), it can be assumed that they occurred in the feed in the form of uncharged molecules. Hence, it is obvious that the separation of 1,3-PD and glycerol was based mainly on the sieving effect while the charge interactions were negligible. The solutes concentrations in the retentate and permeate as well as their retentions in the function of the NF time are shown in Figure 7.

The results obtained in the present study demonstrated that almost all 1,3-PD passed through the membrane used. Indeed, the 1,3-PD retention in the range from 11.2 to −5% (Figure 7a) was recorded. The obtained at the end of the process run the negative values of 1,3-PD retention imply that the system had a higher concentration of the 1,3-PD in the permeate, relative to the retentate [89,90]. It clearly indicates that 1,3-PD tended to penetrate the membrane preferentially. Worthy of note, the low rejection of 1,3-PD does not change its concentration appreciably. Indeed, it has been reported that the concentration of 1,3-PD in the retentate and permeate changed from 12.1 to 12.5 g/L, and from 10.8 to 12.7g/L, respectively.

It was interesting to find that although both 1,3-PD and glycerol were present in the feed solution in the form of neutral molecules, there were notable differences in their rejections. Indeed, in contrast to 1,3-PD, high glycerol retention was observed (Figure 7b). During the first hour of the process run, it was equal to 81% and then it stabilized at the level of about 64%. The noted difference in the glycerol and 1,3-PD retentions can be related to the differences in the weights of the molecules (Table 1). Additionally, it has been reported that the glycerol concentration in the retentate and permeate increased during the NF process run. With regard to the retentate, an increase from 0.2 to 0.5 g/L was noted, while in the permeate the concentration increased from 0.04 to 0.18 g/L. This observation can be explained by the fact that glycerol was retained by the membrane and due to the reduction of feed volume it was concentrated. Consequently, its concentration in the permeate increased with the increased retentate concentration [91].

The importance of this point is that the NF membrane used in the present study ensured the high clarification of 1,3-PD fermentation broths by complete 1,3-PD permeability and a significant rejection of glycerol. Therefore, it can be concluded that the process presented in the current study may be used as a successful and efficient stage in the 1,3-PD separation technology.

#### 3.2.2. Carboxylic Acids and Ethanol

Organic acids which are widely used in the food industry are mainly produced via fermentation processes [92]. Regarding the bioconversion of glycerol to 1,3-PD, the separation of organic acids is aimed at ensuring the high purity of the target product. Moreover, the inhibitory effects of these by-products during the glycerol fermentation processes have been documented [93,94].

The retention mechanism of organic acids during NF is complex since the charge of both solute and membrane depends on the solution pH. As reported before, the skin layer of the membrane used in the present study was made from polyamide (Table 2), which is a hydrophilic polymer and possesses dissociable carboxylic and amine groups (Figure 4). It has been reported that the surface of the membrane is negatively charged when the solution pH is higher than 3.3–4 [88]. The key highlight is therefore that during the NF process of fermentation broths, the membrane surface was negatively charged.

With regards to organic acids, at the feed pH < pKa, they exist in the form of neutral molecules, while at pH > pKa they dissociate into ionic form, as follows [95]:

(8) $$R-H\underset{K_{a}}{\to }R^{-}+H^{+}$$

The pKa values of organic acids present in the broths (Table 1) are lower than the pH of the broth. This indicates that during the NF process they occurred in the charged ionic forms in the feed solution.

The downstream processing of succinic acid reaches more than 80% of the overall production costs [69]. Succinic acid is a dicarboxylic acid, which can exist in the solution in the three following forms: neutral, monovalent, and divalent [52]. At pH values above 7.0 it almost completely dissociates, as follows [66,96]:

(9) $$C_{2}H_{4}C_{2}O_{4}H_{2}↔{\;C}_{2}H_{4}C_{2}O_{4}H^{-}+H^{+}$$

(10) $$C_{2}H_{4}C_{2}O_{4}H^{-}↔{\;C}_{2}H_{4}C_{2}O_{4}^{2-}+H^{+}$$

**Figure 8:**
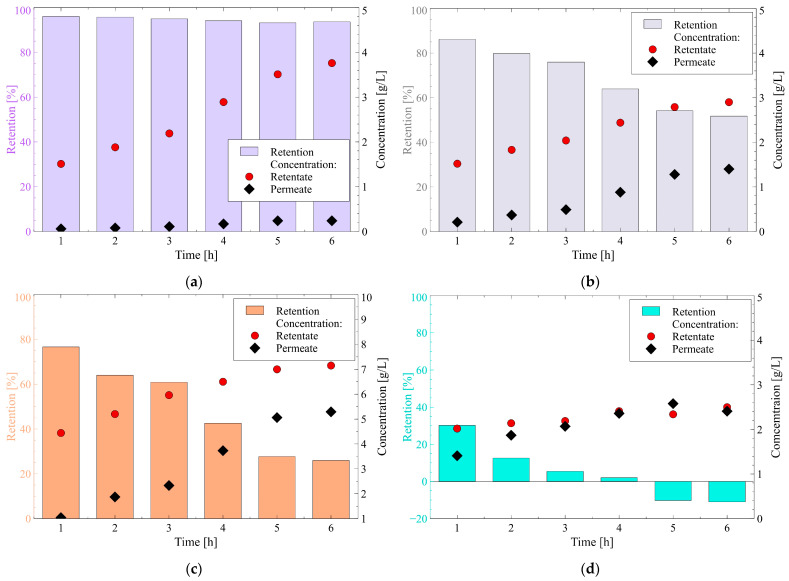

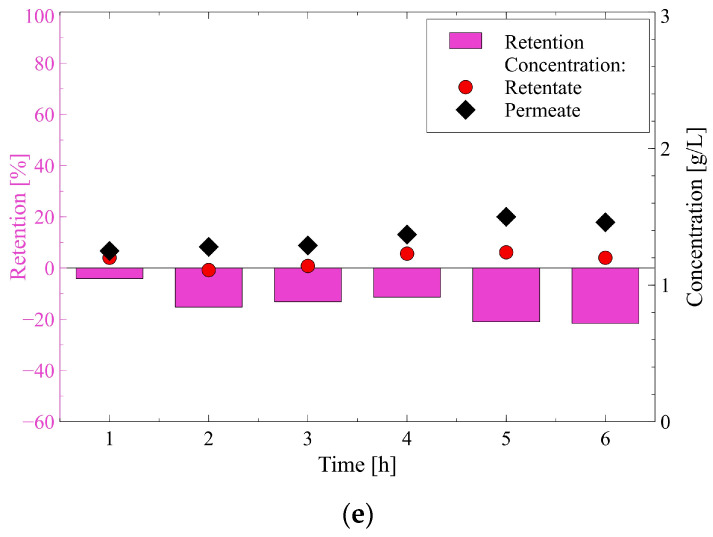
Changes in retention and concentration of the main by-products during the NF process of fermentation broth: (**a**) Succinic acid; (**b**) Lactic acid; (**c**) Acetic acid; (**d**) Formic acid; (**e**) Ethanol. TMP = 1.4 MPa, T = 298 K.

Hence, it can be indicated that succinic acid occurred in the fermentation broth in the form of succinate C_2_H_4_C_2_O_4_^2−^. Figure 8a shows the changes in the succinate retention as well as its concentration in the retentate and permeate during the NF process run. It has been reported that the succinate concentration on both sides of the membrane increased gradually with time. Indeed, the concentration in the retentate increased from 1.5 to 3.8 g/L, while in the obtained permeate it varied from 0.06 to 0.24 g/L. Furthermore, it has been found that although the succinic radius (Table 1) is smaller than the average membrane pore radius (Table 2), the membrane used provides high retention of succinate remaining over 90%. Therefore, the results obtained in the present study demonstrated that the NF membrane ensures the successful separation of succinic acid from 1,3-PD fermentation broths. Since the high fraction of succinic acid existed in the feed in the divalent form, it is apparent that the separation mechanism was based on the Donnan exclusion. The above-presented findings are in agreement with the results of several previous studies [95,97,98,99] wherein high retention of succinic acid during the NF process with the use of various membranes was demonstrated.

Lactic acid may be produced in two ways: biotechnological process and chemical synthesis [16,79]. However, according to [100], 90% of all lactic acid produced worldwide is derived via fermentation processes and the cost of its separation from fermentation broths can reach 50% of the total process costs. Since the pKa of lactic acid (Table 1) is lower than the broth pH (equal to 7), it can be assumed that in the feed, it occurred in the dissociated form CH_3_CHOHCOO^−^, according to the following equation:

(11) $${\mathrm{CH}}_{3}\mathrm{CHOHCOOH}↔{\;\mathrm{CH}}_{3}{\mathrm{CHOHCOO}}^{-}+H^{+}$$

It was observed during the experimental investigation that the lactate concentration in the retentate and permeate increased gradually with the NF process time. Indeed, during the process run the concentration increased from 1.5 to 2.9 g/L and from 0.2 to 1.4 g/L in the retentate and permeate, respectively (Figure 8b). Moreover, a significant reduction of lactate retention during the process has been reported. During the first hour of the process, the retention was equal to 86.2%, while, at the end of the run, the retention of 51.7% was noted. Similar observations have been reported in [101] wherein the NF process of simulated fermentation broth was investigated. According to [50,64,95,102] it can be a consequence of the screening effect which leads to the weakening of electrostatic interactions between the membrane surface and ions CH_3_CHOHCOO^−^ due to the increase in the concentration of particles or counter-ions.

With regards to acetic acid, during the performed NF process, it occurred in the feed in the form of acetate ion CHCOO^−^, according to the following equation [103]:

(12) $${\mathrm{CH}}_{3}\mathrm{COOH}↔{\;\mathrm{CH}}_{3}{\mathrm{COO}}^{-}+H^{+}$$

Similar to butyric and lactic acids, the acetic acid concentration in the retentate and permeate increased gradually with the processing time (Figure 8c). Indeed, at the beginning of the NF, the acetic acid concentration in the retentate equal to 2.2 g/L was noted, while in the end, it increased to 7.1g/L. In turn, in the permeate, an increase in the acetate concentration from 1 to 5.3 g/L was found. Worthy of note, the retention of acetic acid was much lower than that noted for previously reported acids. Results obtained in the present study have demonstrated that at the end of the process run, the retention of acetic acid was equal to about 26%. The poor retention of acetic acid (lower than 40%) was also reported during the separation of 2,3-butanediol fermentation broths with the use of various NF membranes [51].

Currently, formic acid is mostly produced via fermentation processes [104]. The pKa value of formic acid (Table 1) is smaller than the broth pH (equal to 7), hence, it occurred in the feed in an ionic form, as follows:

(13) $$\mathrm{HCOOH}↔{\;\mathrm{HCOO}}^{-}+H^{+}$$

The concentration of formate in the retentate and permeate varied in the range from 2 to 2.2 g/L and from 1.4 to 2.6 g/L, respectively (Figure 8d). Also, in the case of formic acid, the decrease in retention during the process run was noted, showing the impact of the screening effect. Indeed, at the end of the process run, the formate retention of −10% was recorded. Worthy of note, the negative retention of formic acid was also reported in the previously published study [7].

It has been found that in addition to organic acids, ethanol was also a by-product of the glycerol fermentation process. Since the pKa of ethanol is equal to 15.90 (Table 1), it can be indicated that it occurred in the fermentation broths in the form of neutral molecules. Hence, it can be assumed that likewise to glycerol and 1,3-PD, its separation mechanism was based on the sieving effect. As expected, the membrane used in the present study did not ensure the rejection of ethanol. Indeed, during the process, negative retention in the range from −4.1 to −21.7% was noted (Figure 8e). It can be explained by the fact that the Stokes radius and MW of ethanol (Table 1) were much smaller than the membrane average pore size and molecular weight cut-off (Table 2). Hence, the ethanol molecules could easily pass through the membrane. To be complete, it should be mentioned the ethanol concentration in the retentate was equal to about 1.2 g/L, while the permeate was in the range from 1.2 to 1.5 g/L.

Taking into account the above, it can be indicated that the membrane used in the present study allowed us to separate by-products from the target product to varying degrees. Indeed, the separation of succinic acid was almost complete, while the retentions of lactic and acetic acids were only to a certain extent. In turn, the membrane was completely permeable to formic acid and ethanol.

**Figure 9:**
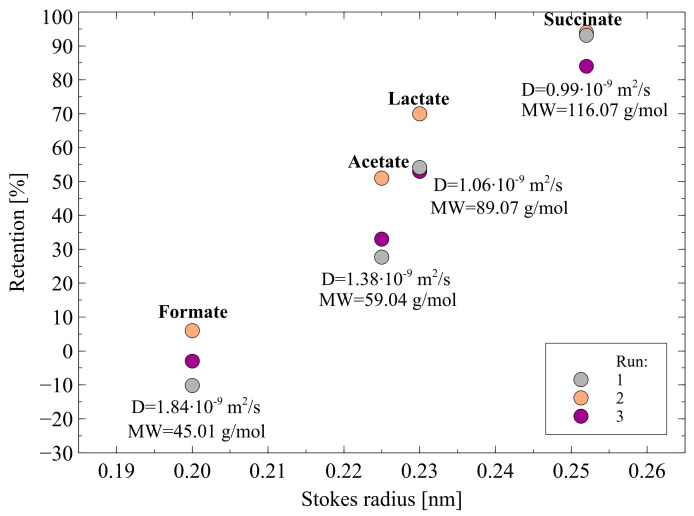
Retention of organic acids in function of Stokes radius during the NF process. TMP = 1.4 MPa, T = 298 K, J = 30 L/m^2^h.

As shown in Figure 9, for each NF process run, the following rejection sequence has been found: succinic acid > lactic acid > acetic acid > formic acid, whereby this relation was true for all the permeate fluxes obtained. Worthy of note, the same trend has been noted in [64], wherein the removal of organic acid salts from simulated fermentation broth with the use of NF45 membrane has been investigated. Based on the data shown in Table 1 and Figure 9, the finding presented in the current study can be explained in several ways:

(i) The highest rejection of succinic acid is related to the fact that it is a diprotic acid, hence, its separation was based on much stronger electrostatic interactions than in the cases of monoprotic acid (lactic, acetic, and formic acids).
(ii) The Stokes radius and MW of charged acids ions: formate, acetate, lactate, and succinate are 0.200 nm and 45.01 g/mol, 0.225 nm and 59.04 g/mol, 0.230 nm and 89.07 g/mol, 0.255 nm and 116.07 g/mol, respectively. It indicates that the highest size ions were retained to a greater extent by the membrane, while the ions with the smallest size were characterized by the lowest retention. This finding indicated that the organic acids retention was based not only on the Donnan exclusion but also on the sieve mechanism.
(iii) For anions with a larger radius, the charge center is farther from the surface and consequently, the electrostatic interactions between anions and negatively charged membrane surface are weaker [38].
(iv) The diffusion coefficients of charged acids ions: formate, acetate, lactate, and succinate are 0.99·10^−9^, 1.06 10^−9^, 1.38·10^−9^, and 1.84 10^−9^ m/s^2^, respectively. Therefore, it can be indicated that the above-presented order of diffusion coefficients is inversely reflected in the rejection sequence. Indeed, ions characterized by the highest diffusion coefficient more easily flow through the membrane.

To sum up, it can be indicated that the retention of organic acids during the NF process with the use of a negatively charged membrane is based on the Donnan effect and sieve mechanism. Indeed, acids retention increases with the charged acids ions valency, Stokes radius, and molecular weight while decreasing with the decrease in the diffusion coefficient.

### 3.3. Separation of Ions

#### 3.3.1. Anions

**Figure 10:**
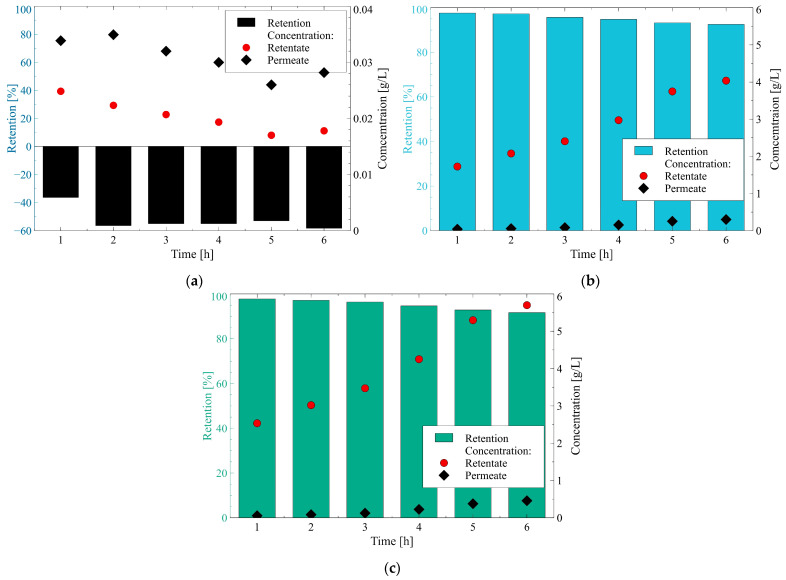
Changes in retention and concentration of the ions during the NF process of fermentation broth: (**a**) Cl^−^; (**b**) SO_4_^2−^; (**c**) PO_4_^3−^. TMP = 1.4 MPa, T = 298 K.

The anions present in the fermentation broths were polyatomic SO_4_^2−^ and PO_4_^3−^ with a tetrahedral shape as well as monoatomic spherical Cl^−^ (Table 1). As indicated above, the surface of the membrane used in the present study, during the separation of 1,3-PD fermentation broths was negatively charged. Moreover, it is necessary to mention that the average size of the membrane pores is similar to the hydrated radius of anions present in the fermentation broths. Hence, it can be assumed that to enter the membrane pores and pass through the membrane, they underwent partial dehydration. The changes in anions retention and concentration during the NF process run are shown in Figure 10.

Anions Cl^−^ have a hydrated radius of 0.332 nm and hydration energy equal to −340 kJ/mol (Table 1). It should be pointed out that the concentration of Cl^−^ was approximately one order of magnitude lower than those of others anions present in the fermentation broths. Indeed, it has been found that Cl^−^ concentration in the retentate and permeate was in the range from 0.017 to 0.025 g/L and from 0.026 to 0.033 g/L, respectively (Figure 10a). It has been found that during the first hour of the NF process run, the retention of Cl^−^ was equal to −36%, and then, it stabilized at the level of about −55%. As mentioned above, the negative values of retention indicate that the compound concentration in the permeate was higher than that reported in the retentate. Negative values of the Cl^−^ retention have been widely recognized during the NF processes of different types of feed with the use of various membranes [105,106,107]. It can be explained by the fact that high rejections of SO_4_^2−^ and PO_4_^3−^ implied that more Cl^−^ passed through the membrane to maintain permeate electro-neutrality [57,108,109]. Additionally, as indicated above anions Cl^−^ are characterized by relatively low hydration energy, hence, they can easily reduce the number of water molecules in the hydration shell and pass through the membrane. Therefore, it can be indicated that the sieve mechanism, pumping effect and dielectric exclusion played an important role in the Cl^−^ separation mechanism.

Anions SO_4_^2−^ are characterized by the hydrated radius and hydration energy equal to 0.379 nm and −1080 kJ/mol, respectively (Table 1). Figure 10b shows the changes in the SO_4_^2^- retention and concentration during the NF run. The SO_4_^2−^ concentration increased with time. Indeed, it has been found that SO_4_^2−^ concentration increased from 1.7 to 4 g/L and from 0.04 to 0.3 g/L in the retentate and permeate, respectively. As expected, the membrane ensured high retention of SO_4_^2−^. Indeed, during the NF process run, retention higher than 92% was obtained. It is important to mention here, that these results are in line with those reported in previous studies [108,110] wherein the high retention (>90%) of SO_4_^2−^ with the use of various NF membranes has been demonstrated.

In turn, anions PO_4_^3−^ have a hydrated radius and hydration energy equal to 0.339 nm and −2765 kJ/mol, respectively (Table 1). As in the case of SO_4_^2-^, a significant increase in the PO_4_^3−^ concentration during the NF process has been found. Indeed, during the experimental run, the PO_4_^3−^ concentration increased from 2.5 to 5.7 g/L and from 0.06 to 0.5 g/L in the retentate and permeate, respectively (Figure 10c). The membrane used in the present study was able to significantly remove PO_4_^3−^ from the fermentation broths. Indeed, during the separation process, a PO_4_^3−^ retention higher than 91% was noted.

The key highlight is therefore that the membrane used in the present study ensured almost total separation of SO_4_^2−^ and PO_4_^3−^ from the target product. As a matter of fact, the obtained permeate was rich in Cl^−^ anions.

**Figure 11:**
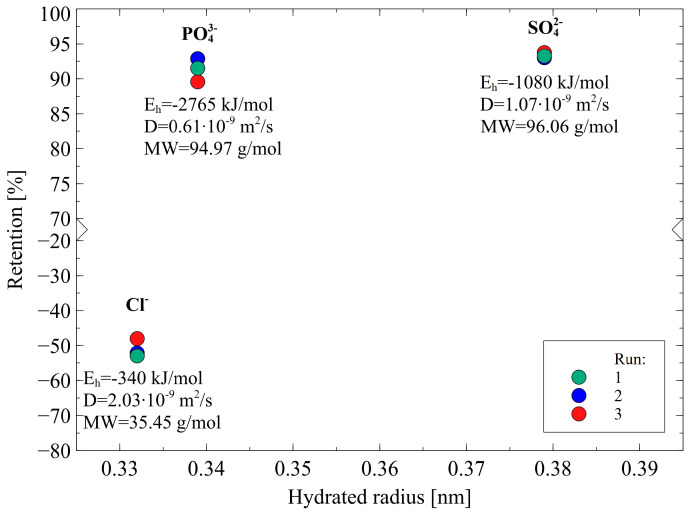
Retention of anions in function of hydrated radius during the NF process. TMP = 1.4 MPa, T = 298 K, J = 30 L/m^2^h.

In this study, anions rejections were evaluated in the following order: SO_4_^2−^ = PO_4_^3−^ > Cl^−^. Based on the data presented in Table 1 and Figure 11, the noted finding can be interpreted in several ways:

(i) The rejection of divalent SO_4_^2−^ and trivalent PO_4_^3−^ anions was higher than that of monovalent ions Cl^−^ due to the fact that they are characterized by higher charge density. This observation indicates that the Donnan effect was the key mechanism of the separation of anions present in 1,3-PD fermentation broths.
(ii) The Stokes radius and MW of anions: Cl^-^, NO_3_^−^, PO_4_^3−^ and SO_4_^2−^ are 0.332 nm and 35.45 g/mol, 0.335 nm and 62 g/mol, 0.339 nm and 94.97 g/mol, 0.379 nm and 96.06 g/mol, respectively. It indicates that the highest retention was obtained for anions that present the highest both radius and MW. Therefore, it can be concluded that the steric hindrance effects also played a significant role in the anion’s separation. It is confirmed by the fact that for anions Cl^−^, negative retention has been reported.
(iii) In the case of ions with a larger radius, the charge center is at a greater distance from the surface and consequently, the electrostatic interactions between ions and the negative membrane surface are weaker [38].
(iv) In light of dielectric exclusion theory, ions Cl^−^ have significantly lower hydration-free energy than PO_4_^3−^ and SO_4_^2−^, hence, they can easily reduce the number of water molecules in the hydration shell:

(14) $$X{\left(H_{2}O\right)}_{n}{}^{m-}↔{\;X}^{m-}+{\mathrm{nH}}_{2}O$$

and consequently, flow easily through the membrane [37].

Based on all experiments performed in this section it can be indicated that for multivalent ions, SO_4_^2−^ and PO_4_^3−^, show the highest retention, which is due to the fact that they have the highest charge density as well as the highest both radius and hydration energy. It clearly demonstrated that the anions separation was based on both the Donnan exclusion, sieving effect, and dielectric exclusion.

#### 3.3.2. Cations

**Figure 12:**
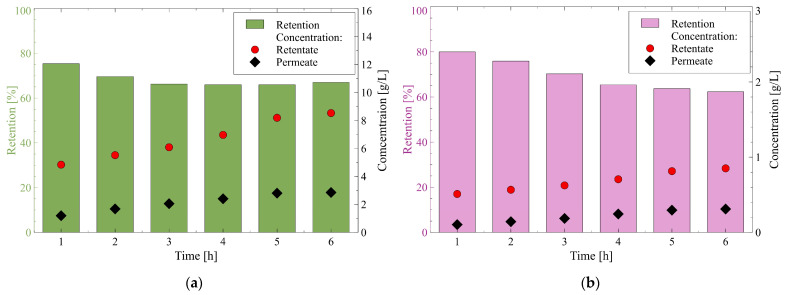

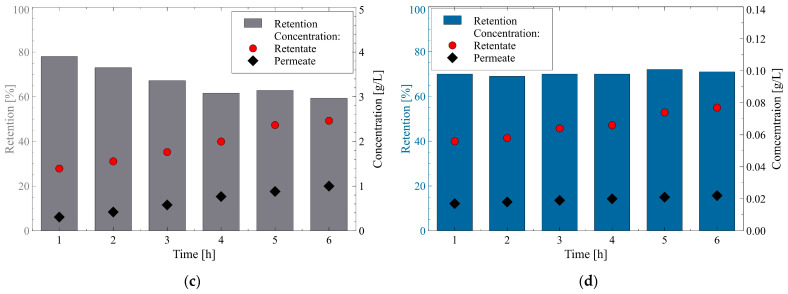
Changes in the retention and concentration of cations during the NF process of fermentation broth: (**a**) Na^+^; (**b**) NH_4_^+^; (**c**) K^+^; (**d**) Ca^2+^. TMP = 1.4 MPa, T = 298 K.

All cations present in the fermentation broths, except ammonium ions NH_4_^+^, are monoatomic and have a spherical shape, while the polyatomic cations NH_4_^+^ have a tetrahedral structure (Table 1). The changes in cations retention and concentration during the NF process run are presented in Figure 12. As expected, the retentions of cations were lower than those obtained for anions. Obviously, it is due to the fact that according to the Donnan exclusion theory (Figure 1b), ions with the opposite charge sign easily pass the membrane. This finding points out that the membrane permeability to cations was higher than to anions. However, likewise, to anions, the hydrated radii of cations present in the fermentation broths (Table 1) were similar to or higher than the average membrane pores size (Table 2). Hence, it can be assumed that to pass the membrane pores, cations underwent partial dehydration. In turn, as has been indicated earlier, cations that have been retained by the membrane could be adsorbed on its surface and, consequently led to a decrease in the membrane performance (Figure 6) and to a reduction in the negative charge of the membrane surface.

Cations Na^+^ are characterized by the hydrated radius and hydration energy of 0.358 nm and −365 kJ/mol, respectively (Table 1). Results presented in Figure 12a demonstrate that during the NF process of fermentation broth, the Na^+^ concentration in the retentate and permeate increased from 4.8 and 8.5 g/L and from 1.2 to 3.5 g/L, respectively. It is very interesting to observe that the retention of Na^+^ in the first hours of the process run decreased and finally, it stabilized at the level of 66%.

Similar observations have been noted for NH_4_^+^ and K^+^ cations (Figure 12b,c). Cations NH_4_^+^ have the hydrated radius and hydration energy of 0.331 nm and −326 kJ/mol, respectively (Table 1). The increase in the NH_4_^+^ concentration in the retentate and permeate from 4.8 to 8.5 g/L and from 1.2 to 3.5g/L, respectively, were noted. Simultaneously, it has been found that NH_4_^+^ retention stabilized at the level of 58%.

With regards to cations K^+^, the hydrated radius and hydration energy are equal to 0.331 nm and −295 kJ/mol, respectively (Table 1). The K^+^ concentration in the retentate increased from 1.4 to 2.5 g/L, while in the permeate an increase from 0.3 to 1 g/L was noted. Noteworthily, the decrease in the retention of K^+^ ions with an increase in the volume concentration ratio was also observed in [63], wherein the production of lactic acid in a bioreactor coupled with NF membranes was studied. In the present study, the K^+^ retention stabilized at the level of 59%. This noteworthy result indicates that the membrane ensured the retention of K^+^ and NH_4_^+^ cations at the same level.

Finally, cations Ca^2+^ have the hydrated radius and hydration energy of 0.412 and −1505 kJ/mol, respectively (Table 1). It was observed during an experimental investigation that among all cations, the concentration of Ca^2+^ was about one order of magnitude lower than those of Na^+^, NH_4_^+^, and K^+^ (Figure 12d). Indeed, the Ca^2+^ concentration varies in the range from 0.05 to 0.07 g/L, while in the permeate from 0.01 to 0.02 g/L. Notably, it has been documented that the stabilized retention of Ca^2+^ was the highest and equal to 72%.

Based on the findings presented above, it can be inferred that cations present in 1,3-PD fermentation broths had different tendencies to penetrate the membrane. However, the highest retention has been noted for Ca^2+^.

**Figure 13:**
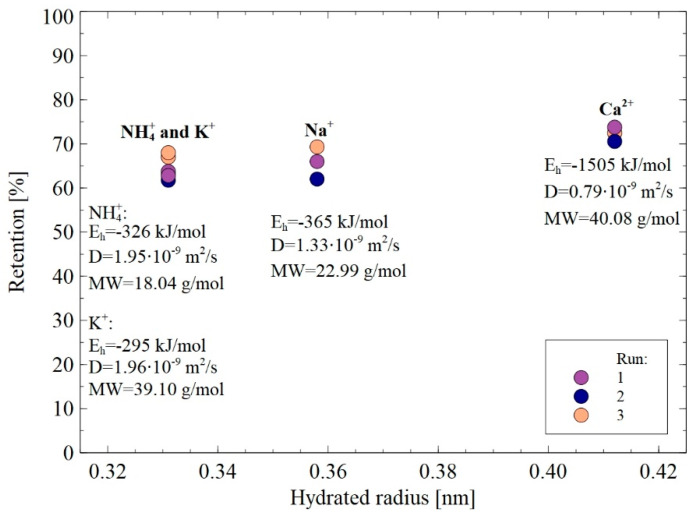
Retention of cations in function of hydrated radius during the NF process. TMP = 1.4 MPa, T = 298 K, J = 30 L/m^2^h.

It has been found that the membrane used in the present study enables the removal of cations to different extents. Indeed, the noted rejection trend of cation was as follows: Ca^2+^ > Na^+^ > NH_4_^+^ ~ K^+^. Based on the data shown in Table 1 and Figure 13, the noted finding can be explained in the following ways:

(i) The rejection of divalent cation Ca^2+^ was higher than that of monovalent ones NH_4_^+^, K^+^, and Na^+^, which can be attributed to the fact that Ca^2+^ is characterized by higher charge density. This result indicates the key role of the Donnan effect during the separation of cations present in 1,3-PD fermentation broths.
(ii) The Stokes radius of cations: NH_4_^+^, K^+^, Na^+^, and Ca^2+^ are 0.331 nm, 0.331 nm, 0.358 nm, and 0.412 nm, respectively. It indicates that the highest retention was obtained for cations characterized by the highest hydrated radius. Therefore, it can be concluded that the steric hindrance effects also played a significant role in the cations separation. It is confirmed by the fact that the same retention degree (59%) has been noted for cations (NH_4_^+^ and K^+^) with the same hydrated radius (0.331 nm). It is necessary to mention that no effect of MW on the retention was recorded, which indicates that with regard to cations separation, the hydrated radius is a more significant parameter.
(iii) The diffusion coefficients of cations: NH_4_^+^, K^+^, Na^+^, and Ca^2+^ are equal to 1.95·10^−9^, 1.96·10^−9^, 1.33·10^−9^, and 0.97·10^−9^ m^2^/s respectively, thus, they are inversely reflected in the rejection sequence. It confirms the thesis that cations with higher diffusion coefficients easily passed through the membrane.
(iv) (The lowest energy hydration, equal to −326 and −295 kJ/mol, have been reported for NH_4_^+^ and K^+^, respectively, hence, they can easily reduce the number of water molecules in the hydration shell (Equation (14)) and consequently, flow easily through the membrane. In turn, multivalent cations such as Ca^2+^ require more energy to lose their hydration shells, so their flow through the membrane was difficult [38].

According to the aforementioned data, it can be pointed out that for multivalent cations Ca^2+^, the highest retention has been noted, which is due to the fact that they have the highest charge density, hydrated radius, and hydration energy and the lowest diffusion coefficient. Therefore, it can be indicated that the cations separation, likewise to the separation of anions, was based on the Donnan exclusion, sieving effect as well as dielectric exclusion.

## 4. Conclusions

The objective of this study was to improve understanding of the separation mechanisms of 1,3-PD fermentation broths components in the NF process. For this purpose, actual broths obtained via the conversion of glycerol were used as a feed. The experiments were carried out by applying the commercial flat-sheet thin-film polyamide NF270 nanofiltration membrane. The separation results were analyzed and discussed in light of the detailed characteristics of both the membrane and the broth components.

The findings obtained in the present study demonstrated that the membrane ensured the high clarification of 1,3-PD fermentation broths by the complete 1,3-PD permeability and significant rejection of such components as succinic acids as well as SO_4_^2−^ and PO_4_^3−^ anions. These results prove that the NF process can be successfully used as a step in the production technology of high-quality 1,3-PD.

With regards to the separation of organic acids, the following rejection sequence has been found: succinic acid > lactic acid > acetic acid > formic acid. A thorough analysis showed that the retention of organic acids during the NF process was based on the Donnan effect and sieve mechanism. Indeed, acids retention increases with the charged acids ions valency, Stokes radius, and molecular weight and decreases with diffusion coefficient. In turn, in the case of anions, the following order retention was determined: SO_4_^2−^ = PO_4_^3−^ > Cl^−^ and Ca^2+^ > Na^+^ > NH_4_^+^ ~ K^+^. It indicated that the ions retention increased with the charge density, hydrated radius, and hydration energy. Based on these findings, it has been concluded that the separation of the ions was based on both the Donnan exclusion, sieving effect, and dielectric exclusion.

## Figures and Tables

**Table 1:** Chemical species in fermentation broths and their associated properties.

Name	Formula	Molecular Weight MW [Da]	Concentration [g/L]	Dissociation Constant pKa [-]	Charge at pH = 7	Diffusion Coefficient D [10^−9^ m^2^/s]	Stokes Radius r_S_/Hydrated Radius r_H_ [nm]	Hydration Free Energy E_h_ [kJ/mol]	Shape
glycerol	C_3_H_8_O_3_	92.09	0.25–0.28	14.40	neutral	0.95 [67,68]	r_S_ = 0.258 [67,68]	NI	tetrahedral
1,3-propanediol	C_3_H_8_O_2_	76.09	12.21–13.45	14.46	neutral	NI	NI	NI	NI
formic acid	CH_2_O_2_	46.05	2.10–2.31	3.84	negative	formate: 1.84 [69]	formate: r_S_ = 0.200 [69]	formate: −347.95 [17]	trigonal and tetrahedral
acetic acid	C_2_H_4_O_2_	60.05	4.25–4.67	4.76	negative	acetate: 1.38 [69]	acetate: r_S_ = 0.225 [70]	acetate: −328.94 [17]	tetrahedral
lactic acid	C_3_H_6_O_3_	90.08	0.59–1.41	3.08	negative	lactate: 1.06 [71]	lactate: r_S_ = 0.230 [71]	lactate: NI	tetrahedral
succinic acid	C_4_H_6_O_4_	118.08	1.14–1.39	4.21 and 5.64	negative	succinate: 0.99 [69]	succinate: r_S_ = 0.252	succinate: NI	NI
ethanol	C_2_H_6_O	46.10	0.89–1.26	15.90	neutral	1.24 [67]	r_S_ = 0.198 [67]	NI	tetrahedral
chloride	Cl^−^	35.45	0.026–0.041	-	negative	2.03 [72,73,74]	r_H_ = 0.332 [75]	−340 [76]	spherical
nitrate	NO_3_^−^	62.00	0.002	-	negative	1.90 [73,74]	r_H_ = 0.335 [75]	−300 [76]	trigonal
phosphate	PO_4_^3−^	94.97	1.926–2.268	-	negative	0.61 [74]	r_H_ = 0.339 [77]	−2765 [76]	tetrahedral
sulfate	SO_4_^2−^	96.06	1.333–1.536	-	negative	1.07 [72,73,74]	r_H_ = 0.379 [75]	−1080 [76]	tetrahedral
ammonium	NH_4_^+^	18.04	0.448–0.545	-	positive	1.95 [73]	r_H_ = 0.331 [75]	−326 [36]	tetrahedral
sodium	Na^+^	22.99	4.451–4.527	-	positive	1.33 [72,73,74]	r_H_ = 0.358 [75]	−365 [76]	spherical
magnesium	Mg^2+^	24.31	0.003–0.010	-	positive	0.71 [72,73,74]	r_H_ = 0.428 [75]	−1830 [76]	spherical
potassium	K^+^	39.10	1.117–1.499	-	positive	1.96 [72,73]	r_H_ = 0.331 [75]	−295 [76]	spherical
calcium	Ca^2+^	40.08	0.011–0.047	-	positive	0.79 [72,74]	r_H_ = 0.412 [75]	−1505 [76]	spherical

NI—no information.

**Table 2:** Main characteristics of the NF membrane used in the present work.

Parameter	Unit	Value
Manufacturer	[-]	DOW-Filmtec
Skin-layer material	[-]	polyamide
Molecular weight cut-off	[Da]	200–300
Average pore radius	[nm]	0.43
MgSO_4_ rejection	[%]	97
NaCl rejection	[%]	50
Contact angle	[°]	54.8
Maximal pressure	[MPa]	4.1
Maximal temperature	[°C]	45
pH range	[-]	2–11
Isoelectric point	[-]	4

## Data Availability

Not applicable.
